# Construction of recombinant baculoviruses expressing hemagglutinin of H5N1 avian influenza and research on the immunogenicity

**DOI:** 10.1038/srep24290

**Published:** 2016-04-11

**Authors:** Jingping Ge, Qi An, Dongni Gao, Ying Liu, Wenxiang Ping

**Affiliations:** 1 Key Laboratory of Microbiology, College of Life Science, Heilongjiang University, Harbin, China

## Abstract

Recombinant baculoviruses with different promoter and regulatory elements were constructed to enhance the expression of target protein and boost the efficacies of avian influenza vaccine. *Hemagglutinin* gene was cloned into the baculovirus transfer vectors driven by cytomegaloviru (CMV) and White spot syndrome virus immediate-early promoter one (WSSV ie1) promoter respectively, with different regulatory elements. The recombinant baculoviruses were directly used as vaccines to immunize specific pathogen-free chickens. The protein expression levels of recombinant baculoviruses BV-S-HA and BV-S-ITRs-HA were respectively 2.43 and 2.67 times than that of BV-S-con-HA, while the protein expression levels of BV-A-HA and BV-A-ITRs-HA were respectively 2.44 and 2.69 times than that of BV-S-con-HA. Immunoglobulin G (IgG) antibody levels induced by BV-A and BV-S series recombinant baculovirus were significantly higher than the commercialized vaccine group (*P* < 0.05). Among the groups with same promoter, the IgG antibody levels induced by the baculovirus containing regulatory elements were significantly higher than control group. Additionally, the immune effects induced by BV-A series recombinant baculoviruses with WSSV ie1 promoter were significantly stronger than the BV-S series recombinant baculoviruses with CMV promoter. The avian influenza vaccine prepared based on baculovirus vector can simultaneously stimulate the humoral and cellular immune responses.

The highly pathogenic avian influenza caused by influenza A H5N1 virus (Avian ingluenza virus, AIV), is an acute and highly contagious disease^1^. The highly pathogenic avian influenza virus H5N1 not only infects poultry but also can break the barrier between species, pose substantial threat to mammals and humans^2^. Therefore, how to control the epidemic has become one of the world’s most pressing problems. Currently, the main way to prevent and control the infection is immunized with efficient vaccination. The most commonly used vaccine is the inactivated virus vaccine, however, it has shortcomings^3^,^4^. For instance, the preparation of it needs a large amount of chicken embryos, large injection dose and long preparation period^5^. AIV subunit vaccine can provide effective immune protect, but its purification process is complex with high production costs^6^. Additionally, DNA vaccine is easy to be made into multivalent vaccine, but the expression of target protein is still not satisfactory^7^. Thus, it is imperative to research novel AIV vaccines.

AIV genome can be divided into eight segments of RNA, encoding ten kinds of proteins^8^. The pathogenicity of AIV is affected by many factors; hemagglutinin (HA) is one of the vital factors. As the major protective antigen of avian influenza virus, HA performs a vital role in its antigenicity and pathogenicity^9^. HA located in the posterior capsule, capable of agglutinating the red blood cells, stimulate body to produce neutralization antibody. Crawford research team successfully expressed HA via the baculovirus expression system and prepared AIV subunit vaccine to immunized SPF chickens^10^. It turned out that HA antigen can elicit efficient immune response of trial chickens.

As a promising vaccine vector, baculovirus is endowed with many advantages such as its good biosafety, the large-capacity of foreign genes and the simultaneous expression of multiple target genes^11^. Nevertheless, the transduction efficiency and transgene expression levels are not satisfactory which restrict its further application^12^,^13^. Promoter is one of the major factors that affect the expression of recombinant baculovirus, and a stronger promoter can enhance the expression efficiency of exogenous genes. White spot syndrome virus immediate-early promoter one (WSSV ie1) promoter and cytomegalovirus (CMV) promoter are both recognized as strong promoters due to their respective advantages of driving exogenous gene expression efficiently in mammalian cells and insect cells^14^. Furthermore, the woodchuck hepatitis virus post-transcriptional regulatory element (WPRE) and adeno-associated virus inverted terminal repeats (ITRs) play vital roles in improving the expression efficiency of a target gene and extending the expression time^15^,^16^,^17^. ITRs were added on both sides of the promoter expression cassettes. WPRE was inserted in the 3′UTR region of the target gene.

In the study, baculovirus based vaccine was prepared which drived by different promoters with regulatory elements to further optimize the baculovirus expression vector and enhance the efficiency of its infectivity.

## Results

### Western blotting of HA protein in chicken embryo fibroblast cells

**Figure 1:**
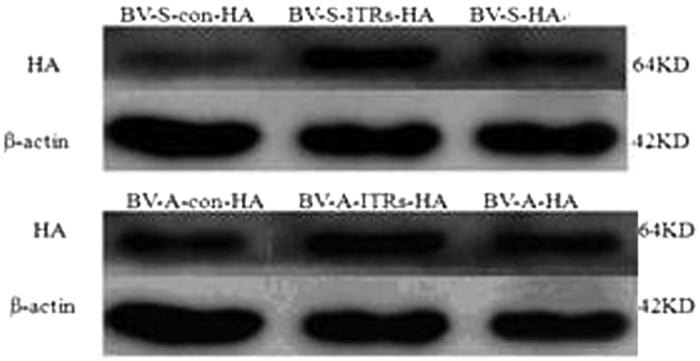
Western blotting of HA protein in chicken embryo fibroblast cells. HA levels in the cell lysates were detected by Western blotting analysis and no band was observed in the control (β-actin), which confirmed successful expression of the target antigenic protein HA. HA and β-actin were 64 KD and 42 KD, respectively. BV-S series (BV-S-HA, BV-S-ITRs-HA and BV-S-con-HA) refers to the recombinant baculovirus start by CMV, containing different regulatory elements of BV-S-HA (WPRE), BV-S-ITRs-HA (WPRE, ITRs), BV-S-con-HA; BV-A series (BV-A-HA, BV-A-ITRs-HA and BV-A-con-HA) refers to the recombinant baculovirus start by ie1, containing different regulatory elements of BV-A-HA (WPRE), BV-A-ITRs-HA (WPRE, ITRs), BV-A-con-HA.

**Figure 2:**
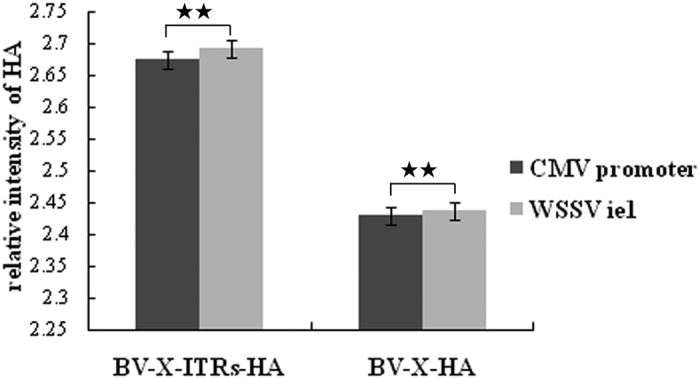
The relative intensity of target protein HA mediated by baculovirus with different promoters. The black bar (■) was the relative intensity of HA driven by CMV promoter; the gray bar (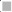)was the relative intensity of HA driven by ie1 promoter; BV-X-HA refers to the recombinant baculovirus with WPRE regulatory elements driven by CMV and ie1 promters, respectively; BV-X-ITRs-HA refers to the recombinant baculovirus with WPRE and ITRs three regulatory elements drives by CMV and ie1 promters, respectively; BV-X-con-HA refers to the recombinant baculovirus without any regulatory elements driven by CMV and ie1 promters, respectively. **P < 0.01.

The BV-S-HA Series (BV-S-HA, BV-S-ITRs-HA, BV-S-con-HA) and BV-A-HA Series recombinant baculovirus (BV-A- HA, BV-A-ITRs-HA, BV-A-con-HA) were used to infect the chicken embryo fibroblast cells, and HA levels in the cell lysates were detected by Western blot analysis. As a result, a band was detected at a molecular weight of approximately 64 kDa, and no band was observed in the control (β-actin), which confirmed successful expression of the target antigenic protein HA (Figs 1 and 2).

### The lymphocyte proliferation effect

**Figure 3:**
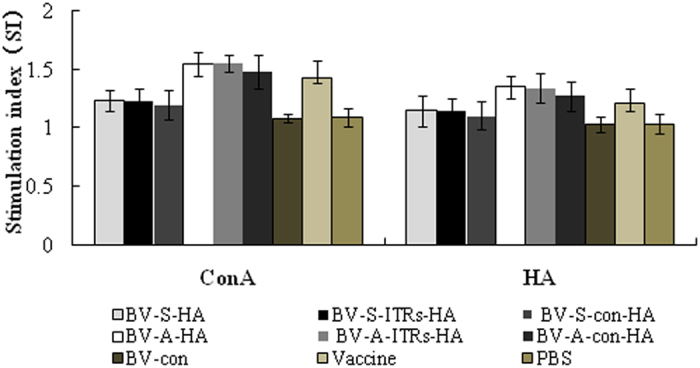
The proliferation response of chicken peripheral blood lymphocyte cells. ConA and HA protein were used to stimulate the proliferation response. The stimulation index of different regulatory elements and different promoters were compared. BV-S series (BV-S-HA, BV-S-ITRs-HA and BV-S-con-HA) refers to the recombinant baculovirus started by CMV, containing different regulatory elements of BV-S-HA (WPRE), BV-S-ITRs-HA (WPRE, ITRs), BV-S-con-HA; BV-A series (BV-A-HA, BV-A-ITRs-HA and BV-A-con-HA) refers to the recombinant baculovirus started by ie1, containing different regulatory elements of BV-A-HA (WPRE), BV-A-ITRs-HA (WPRE, ITRs), BV-A-con-HA. BV-con refers to baculovirus without any target gene, vaccine refers to immunized with commercial inactivated AIV vaccines and PBS refers to control which immunized with PBS. The stimulation index of BV-A series group started by WSSV ie1 promoter significantly higher than BV-S series droved by CMV promoter and the vaccine group. As for the group containing the same promoter, baculovirus with WPRE and ITRs regulatory elements were significant higher than baculovirus not containing any element.

Chicken peripheral blood lymphocyte proliferation results showed that the peripheral blood lymphocytes proliferate significantly stimulated by HA. The stimulation index of BV-A series group started by WSSV ie1 promoter reach up to 1.349 which significantly higher than BV-S series droved by CMV promoter (1.145) and the vaccine group (1.205). As for the group containing the same promoter, baculovirus with WPRE and ITRs regulatory elements were significant higher than baculovirus not containing any element (*P* < 0.05) (Fig. 3).

### Analyses of HA antibody IgG

**Figure 4:**
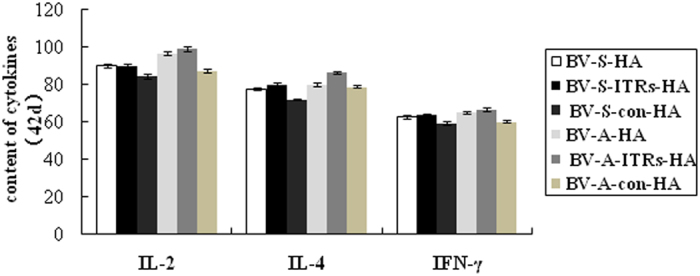
The changes of IgG antibody induced by recombinant baculovirus with different regulatory elements (42d). BV-S-HA and BV-A-HA refers to the recombinant baculovirus with WPRE regulatory element driven by CMV and ie1 promoters respectively; BV-S-ITRs-HA and BV-A-ITRs-HA refers to the recombinant baculovirus with WPRE and ITRs regulatory elements driven by CMV and ie1 promoters, respectively; BV-S-con-HA and BV-A-con-HA refers to the recombinant baculovirus without any regulatory elements driven by CMV and ie1 promoters, respectively.

The results revealed that the six recombinant baculovirus enable to stimulate chickens to generate IgG antibodies, the antibody levels gradually increased along with the extension of time (Fig. 4). While the control group barely changed. The antibody levels of IgG induced by BV-A series recombinant baculovirus and BV-S series recombinant baculovirus were significantly higher than that of the commercialized vaccine group and the control group (*P* < 0.05). Among the groups with same promoter, the IgG antibody levels induced by the baculovirus containing WPRE and ITRs regulatory elements were significantly higher than that of the control.

### Analyses of IL-4, IL-2 and IFN-γ cytokines

**Figure 5:**
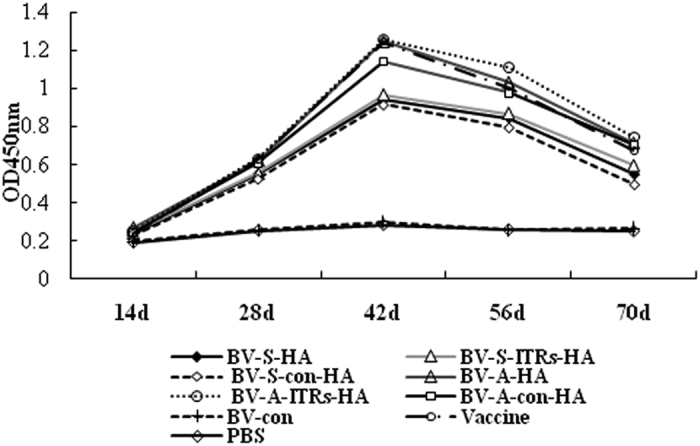
The contents of cytokines induced by recombinant baculovirus with different promoters (ng/L). Black diamond with solid line (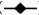) refers to BV-S-HA, open diamond with dash line (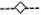) refers to BV-S-con-HA, open circle with dash line (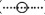) refers to BV-A-ITRs-HA, cross with dash line (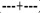) refers to BV-con, open diamond with solid line (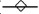) refers to PBS, open triangle with gray line (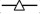) refers to BV-S-ITRs-HA, open triangle with solid line (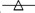) refers to BV-A-HA, open square with solid line (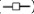) refers to BV-A-con-HA and open circle with dash line (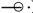) refers to vaccine. BV-S series refers to the recombinant baculovirus start by CMV, containing different regulatory elements of BV-S-HA (WPRE), BV-S-ITRs-HA (WPRE, ITRs), BV-S-con-HA; BV-A series refers to the recombinant baculovirus start by ie1, containing different regulatory elements of BV-A-HA (WPRE), BV-A-ITRs-HA (WPRE, ITRs), BV-A-con-HA.

The cytokine detection results demonstrated that cytokine levels of each group reached the maximum value two weeks post-immunization (Fig. 5). The results revealed that the immune effects induced by BV-A series recombinant baculoviruses with WSSV ie1 promoter were significantly stronger than the BV-S series recombinant baculoviruses with CMV promoter.

## Discussion

In the study of genetically engineered AIV vaccine, many scholars utilize baculovirus as vector of vaccine. There were researches on purifying HA protein of avian influenza virus via baculovirus expression system and using it as subunit vaccine to immunize with chickens^18^. Bethanie and Wickinson prepared different subtypes of recombinant adjuvant vaccine based on baculovirus, which were turned out to provide 100% immune protection to experimental animals^19^. In addition, many researchers have conducted research on avian influenza DNA vaccine. However, the study found that the antibody level induced by DNA vaccines is relatively weak since the immune effects of DNA vaccines will be affected by the eukaryotic expression vector. Meanwhile, different immune pathways and immunization dose will affect the immune effect. Thus, it is imperative to research more efficient AIV vaccine.

Utilizing the recombinant baculovirus directly as vaccine to immunize with poultry has distinct advantages. Compared with traditional attenuated vaccine and genetically engineered live vector vaccine, the baculovirus do not replicate in avian cells, so it endowed with good biosecurity^20^,^21^,^22^,^23^,^24^. Furthermore, the recombinant baculovirus can form virus-like particle which was similar to the natural virion of AIV so as to simulate the natural infection and mediate HA expressing in the mammalian cells.

In this study, we are focus on searching for more efficient promoter and enhancer sequences to improve the expression of target protein. Firstly, the recombinant vectors were constructed via dual-expression baculovirus system. The two expression cassettes on the baculovirus could express independently, one of it containing pPolH promoter could started gene expressed in sf9 cells. Another expression cassette containing either CMV promoter or WSSV ie1 promoter can drive the expression of *HA* gene and the regulatory element. Studies have shown that integrated the woodchuck hepatitis virus regulatory (WPRE) in the original gene of the 3′non-coding region can increase the expression of target gene. And add adeno-associated virus inverted terminal repeats (ITRs) on both sides of exogenous gene can maintain a high level of exogenous gene expression and prolong the time of expression. In our study, we demonstrated that WPRE and ITRs regulatory elements play a vital role in enhance and extend the expression of HA.

Researches showed that the effect of immunization is closely related to the injection dose and programs of immunization. The antibody levels of chickens would not satisfactory if the immunization dose is not enough. Yet, the overdose of immunization would lead to death of chickens. The baculovirus expression vectors used in this study has a high bio-security. The dose of immunization was studied pre-experiment. Therefore, we determine the immunizing dose is 1.0 × 10^9^ pfu. Results show that the experimental group could generate antibodies induced by the recombinant baculovirus and provide protection to chickens. But the overall antibody levels are still not satisfactory for our requirement. Therefore, the immunization program should further optimized to enhance the immune effect in subsequent trials.

## Materials and Methods

### Ethics Statement

All animal experiments were carried out in accordance with the Guidelines for Animal Experiments of National Institute of Infectious Diseases (NIID). Experimental protocols were reviewed and approved by the Animal Ethics Committee of Harbin Veterinary Research Institute of the Chinese Academy of Agricultural Sciences (CAAS) and the Animal Ethics Committee of Heilongjiang Province (SYXK (H) 2006-032). Chickens were housed in sterile isolator separately and provided with standard food and water. The health condition was monitored every day.

### Plasmids and Cells

Plasmid pGDN-HA, pS, pS-ITRs, pS-con, pAQ-con-eGFP, pAQ-eGFP, pAQ-ITRs-eGFP were previously prepared by the laboratory. The chicken embryo fibroblast cells and *Sf*9 insect cells were prepared by the laboratory.

### Construction of recombinant baculoviral vectors

*HA* target gene was amplified from pGDN-HA by PCR with a pair of specific primer HA-F: 5′CGCGGGCCCATGGAGAAAATAGTGCTTCTTCTTG (an *Apa* I site was introduced); HA-R: 5′GGCGGGCCCTCA*ATGATGATGATGATGATG*ACT-TAAATGCAAATTCTGCATTGTA (an *Apa* I site was introduced). HA-R contains His tag. The PCR products of *HA* was inserted into the vector pMD18-T vector and sequenced. The correct recombinant plasmid was identified as pT-HA. pT-HA and pS, pS-ITRs, pS-con were digested with *Apa* I restriction endonuclease to generate recombinant plasmids pS-HA, pS-ITRs-HA, pS-con-HA droved by CMV promoter. Then the *eGFP* gene of pAQ-con-eGFP, pAQ-eGFP, pAQ-ITRs-eGFP was substitute with *HA* gene. The identified recombinant plasmids promoted by WSSV ie1 were named pA-HA, pA-ITRs-HA, pA-con-HA.

### Transduction and proliferation of recombinant baculovirus

Plasmids pS-HA, pS-ITRs-HA, pS-con-HA, pA-HA, pA-ITRs-HA, pA-con-HA were transformed into *E. coli* DH10 Bac competent cells which were prepared by the SEM method. Extracted bacmid DNA of positive colonies with alkaline lysis approach. The recombinants were confirmed as rBac-S-HA, rBac-S-ITRs-HA, rBac-S-con-HA, rBac-A- HA, rBac-A-ITRs-HA, rBac-A-con-HA. The baculovirus transfer vectors were separately transfected into *Sf*9 insect cells previously prepared by the laboratory.

The viruses were harvested 72 h post transfected and then further infected into *Sf*9 insect cells with several rounds to get higher titers. Then, the viral genome was extracted and amplified to confirm that the target genes were inserted into the recombinant baculovirus correctly.

### Expression of HA protein in chicken embryo fibroblast cells

The chicken embryo fibroblast cells were prepared by the laboratory. The recombinant baculoviruses BV-S-HA, BV-S-ITRs-HA, BV-S-con-HA, BV-A- HA, BV-A-ITRs-HA, BV-A-con-HA at a multiplicity of infection (MOI) of 100 were separately infected to the chicken embryo fibroblast cells at a density of 5 × 10^5^ cells/mL. The cells were lysed and harvested 48 h post infection for SDS-PAGE electrophoresis and Western blot analysis. The Rabbit anti-histidine tag antibody was used as the primary antibody and the horse radish peroxidase (HRP)-labeled goat anti- rabbit IgG used as the secondary antibody.

### Assessment of Chicken immune effect

#### The immune plan

Fourteen-day old specific pathogen free (SPF) chickens were raised at randomly divided into eight groups with eight chickens in each group. Groups A was set for CMV promoter experimental group which respectively immunized with (A1: BV-S-HA, A2: BV-S-ITRs-HA, A3: BV-S-con-HA). Groups B was set for WSSV ie1 promoter experimental group which respectively immunized with (B1: BV-A-HA, B2: BV-A-ITRs-HA, B3: BV-A-con-HA). Group C was control baculovirus experimental group without the target gene. The chickens in group D was immunized with commercial inactivated AIV vaccines. Group E was set for the control which immunized with PBS. Chickens in groups A, B and C were injected with 10^9^ pfu recombinant baculovirus per chicken; and chickens in group D were immunized with 0.2 mL vaccine per chicken. Chickens of all the vaccinated groups were boosted with the corresponding vaccines at the same dosage 14 days after the first immunization. Blood samples were obtained from six immunized chickens selected at random from each group after 14, 21, 28, 35, 42, 49, 56 and 70 days; the blood was separated by centrifugation, and the serum was collected for analysis.

#### The lymphocyte proliferation of chickens

The peripheral bloods of immune chickens were collected regularly to prepare chicken peripheral T lymphocytes. ConA and HA protein were used to stimulate the effect of lymphocyte proliferation. The peripheral blood lymphocytes were inoculated into 96-well plates with 100 μL per well. The experimental group added RPMI1640 medium containing HA protein. The positive control wells added RPMI1640 medium containing ConA. The negative control wells only added RPMI1640 culture medium, each group set up three replicate wells. Cells were cultured at 37 °C 5% CO_2_. Incubated post 44 h, added 20 μL 5 mg/mL MTT solution to each hole and incubated in the dark for 4 h. Detected at a wavelength of OD 490 nm values and calculated for the stimulation index (SI) of each group.

#### ELISA for detection of anti-HA antibodies

The serum antibody levels of the immunized chickens were detected by ELISA kit. In accordance with the instructions of the AIV antibody detection kit, the negative control, positive control and samples were added to the lath respectively, and the OD value was measured at a wavelength of 450 nm.

#### Analyses of IL-4, IL-2 and IFN-γ cytokines

The content of IL-2, IL-4 and IFN-γ cytokines were detected by ELISA Kit of interleukin 4, interleukin 2 and interferon γ which purchased by Shanghai Du Bridge Trading Company. In accordance with the instructions of the ELISA Kit, the standard linear regression curves were plotted and concentration of IL-2, IL-4 and IFN-γ cytokines were detected.

### Statistical analysis

The mean difference between samples data were compared and presented as X ± SD by SPSS Statistics19 software. Statistical significance was assessed by one-way analysis of variance, *P* < 0.05 was considered significant.

## Additional Information

**How to cite this article**: Ge, J. *et al.* Construction of recombinant baculoviruses expressing hemagglutinin of H5N1 avian influenza and research on the immunogenicity. *Sci. Rep.* **6**, 24290; doi: 10.1038/srep24290 (2016).
